# Reproductive coercion in Uttar Pradesh, India: Prevalence and associations with partner violence and reproductive health

**DOI:** 10.1016/j.ssmph.2019.100484

**Published:** 2019-11-20

**Authors:** Jay G. Silverman, Sabrina C. Boyce, Nabamallika Dehingia, Namratha Rao, Dharmoo Chandurkar, Priya Nanda, Katherine Hay, Yamini Atmavilas, Niranjan Saggurti, Anita Raj

**Affiliations:** a Center on Gender Equity and Health, Division of Global Public Health, University of California San Diego School of Medicine, La Jolla CA, USA; b Sambodhi Research and Communications Pvt. Ltd., Uttar Pradesh, India; c Bill and Melinda Gates Foundation in New Delhi, India; d Bill and Melinda Gates Foundation in Seattle, WA, USA

**Keywords:** Reproductive coercion, Intimate partner violence, India, Unintended pregnancy, Contraception

## Abstract

Increasing modern contraceptive use and gender equity are major foci of the recently ratified Sustainable Development Goals for 2030 and the Government of India. Coercion and sabotage by husbands and in-laws to inhibit women's access, initiation, continuation, and successful use of modern contraception methods (i.e., *reproductive* coercion) may contribute to low usage rates and unintended pregnancy in India; however, little is known about the extent of this problem. The current study assesses the prevalence of reproductive coercion, both husband and in-law perpetrated, among a large population-based sample. Data were collected from currently married women of reproductive age (15–49 years; N = 1770) across 49 districts of Uttar Pradesh as part of an evaluation of a broad effort to improve the public health system in the state. Dependent variables included modern contraceptive use in the past 12 months, unintended pregnancy, and pregnancy termination. Independent variables included ever experiencing reproductive coercion (RC) by a current husband or in-laws and lifetime experience of physical and sexual intimate partner violence (IPV) by a current husband. Approximately 1 in 8 (12%) women reported ever experiencing RC from their current husbands or in-laws; 42% of these women reported RC by husbands only, 48% reported RC by in-laws only, and 10% reported RC by both husbands and in-laws. Among women experiencing RC, more than one-third (36%) reported that their most recent pregnancy was unintended; these women had 4 to 5 times greater odds of unintended pregnancy and a more than 5 times decreased likelihood of recent use of modern contraceptives than women not experiencing RC, after accounting for effects of demographics and physical and sexual IPV. Scalable and sustainable interventions in both clinical and community settings are needed to reduce RC, a potentially key factor in effective strategies for improving women's reproductive autonomy and health in India and globally.

## Highlights

• 1 in 8 women in Uttar Pradesh report reproductive coercion from husbands or in-laws.
• Husbands and in-laws were responsible for RC in similar measure (42% and 48%).
• Experiencing RC related to 4–5x greater odds of recent unintended pregnancy.
• Experiencing RC related to 5x decreased odds of recent contraceptive use.
• Reducing RC is key to improving women's reproductive autonomy and health.

## Introduction

Low rates of modern contraceptive use continue to plague the majority of low- and middle-income countries (LMICs). LMICs with the lowest contraceptive prevalence rates also suffer from related high levels of maternal and neonatal mortality as well as gender inequity (Ahmed et al, 2010, 2012; Filippi et al., 2006). Increasing modern contraceptive use and gender equity are major foci of the recently ratified Sustainable Development Goals (SDGs) for 2030 (WHO, 2015). Uttar Pradesh, with a population of 200 million, is the most populous state in India, and lacks sufficient contraceptive coverage, with only 1 in 5 women in the state using any form of modern contraception (AHS 2012–13). Although two-thirds of women in Uttar Pradesh with 2 children report desire to have no additional pregnancies, the current fertility rate remains at 3.8 (IIPS and ICF, 2017a). Unmet need is greatest among Muslim, poor, and adolescent married women (IIPS and ICF, 2017a). However, the greatest social barrier to contraceptive use in Uttar Pradesh is husband opposition, with 1 in 6 women reporting this as the reason that they cannot currently use a modern contraceptive method.

Intimate partner violence (IPV) from husbands is associated with contraceptive failure in India and is a consistent risk factor for unintended pregnancy in other countries where this association has been examined (Cripe et al., 2008; Gao, Paterson, Carter, & Iusitini, 2008; Pallitto et al., 2013; Silverman, Gupta, Decker, Kapur, & Raj, 2007; Stephenson, Koenig, Acharya, & Roy, 2008). Women across India experience high rates of IPV from husbands, with approximately 1 in 3 ever-married women reporting physical, sexual, or emotional violence from their spouse (IIPS and ICF, 2017b). However, unlike many other LMIC contexts, women who experience IPV in India are more likely than other women to report that they were using a form of modern contraception at the time they most recently became pregnant (Raj & McDougal, 2015). This combination of indicators points to two critically important realities for women in India: husband opposition often prevents women's attempts to use contraceptives, and husband abuse prevents successful use of contraceptives (i.e., contributes to contraceptive failure).

There is a growing body of evidence on the role of husbands and in-laws in women's reproductive decision making in India. (Barua & Kurz, 2001; Ghule et al., 2015; Mishra et al., 2014). Recent research has identified specific mechanisms by which male partners directly interfere with women's attempts to use contraceptives, including specific forms of coercion and sabotage to inhibit women's access, initiation, continuation, and successful use of modern methods; these mechanisms have been collectively labelled *reproductive coercion (RC)* (McCauley et al., 2017; Miller et al., 2014; Miller et al., 2010). Reproductive coercion is associated with IPV but has also independently predicted contraceptive use and unintended pregnancy in multiple studies that assessed the relative associations of each of these forms of gender-based violence with reproductive outcomes (Stephenson et al., 2008; Miller et al., 2010; Moore, Frohwirth, & Miller, 2010; Miller et al., 2014; Kazmerski et al., 2015)

Consensus regarding the critical and mechanistic role of RC in unintended pregnancy and contraceptive nonuse is growing, with recent guidelines published by the World Health Organization (WHO) identifying RC as a key aspect of gender-based violence to be assessed and considered by health care personnel, particularly in family planning settings (WHO, 2017). Successful models of addressing RC to improve women's reproductive autonomy (i.e., ability to make and enact decisions regarding pregnancy and childbirth) have included brief counselling of women on use of contraceptives that may be used without husband detection (Miller et al., 2016; Tancredi et al., 2015). Population-based studies on the prevalence and nature of this important phenomenon outside of the United States are needed, as are studies that elucidate the roles of RC in women's contraceptive use, unintended pregnancy, or pregnancy termination (i.e., abortion) outside this high-income context. Understanding the independent effects of the related constructs of IPV and RC is also required, as such data may directly inform health care guidelines for screening women at high risk for these adverse reproductive outcomes as well as intervention programming to reduce risk of these outcomes. Conceptions of RC in previous studies have been limited to coercion and interference from male partners; based on several previous studies on the important role of in-laws in both household abuse of their daughters-in-law and decision making regarding reproductive health in India (Fernandez, 1997; Raj et al., 2011; Kamimura et al., 2015; Silverman et al., 2016a, 2016b), extension of assessment of RC among women in India to include in-law involvement is strongly indicated.

The current study will assess the prevalence of RC, both husband and in-law perpetrated, among a large population-based sample of women in Uttar Pradesh, India. Independent effects of IPV (both physical and sexual) and RC on contraceptive use, unintended pregnancy, and abortion will also be examined. Beyond being major objectives of the SDGs for 2030, reducing gender-based violence and increasing use of modern contraceptives are stated priorities for the Indian national public health system (Government of India and National Heath Mission, 2017, Government of India and National Heath Mission, 2018). Findings of the current study may inform public health programs and policies to achieve both of these objectives in the high-need context of Uttar Pradesh as well as other LMIC settings.

## Materials and methods

### Study design

Data utilized for the current study were collected as part of an evaluation of a broad effort to improve the public health system by the Government of Uttar Pradesh and the Bill and Melinda Gates Foundation. The data utilized were collected via the second of two surveys regarding topics related to family planning administered to a population-based sample of households across the state of Uttar Pradesh. Neither partner violence nor reproductive coercion were addressed in the programmatic efforts assessed. All districts of the state (n = 75) were ranked on a composite index comprised of maternal mortality ratio (MMR), percentage of institutional deliveries, infant mortality rate, percentage of children aged 12–23 months who were fully immunized, total fertility rate, and modern contraceptive prevalence rate. The 25 districts ranked lowest based on this composite index were designated high priority districts (HPDs) by the Government of Uttar Pradesh (Bhawan, 2013a, Bhawan, 2013b). A multistage sampling design was employed to obtain a population-based sample of currently married women of reproductive age (15–49 years), inclusive of oversampling from the 25 HPDs of Uttar Pradesh. At the first stage, 4 blocks (areas representing approximately 100,000 residents; range of approximately 10–30 blocks per district) were selected within each HPD—those with highest (2 blocks) and lowest (2 blocks) percentages of facility-based (vs. home) deliveries. These 100 blocks (4 blocks in each of 25 districts) were then matched to another 100 blocks from across the remaining districts of Uttar Pradesh based on socioeconomic data inclusive of caste, religion, household wealth, and adult literacy; data utilized for this matching process were drawn from the 2011 Government of India Census. This activity resulted in inclusion of blocks within an additional 24 districts. At the second level, within each of the 200 selected blocks, 3 Accredited Social Health Activist (ASHA; i.e., community health worker) catchment areas consisting of approximately 1000 households (there are approximately 150–450 ASHA areas per block) were randomly selected, resulting in a total sample of 600 ASHA areas. At the third level, a census of all the households from each selected ASHA area was conducted to identify women who were married and of reproductive age (15–49 years). Of the listing of eligible women identified, 4 were randomly selected from each ASHA catchment area. Out of the 2400 originally selected women, a total of 2222 women (92.6%) consented to and completed the first survey conducted in 2014. Using contact information collected at the first wave, participating women continuing to reside in the selected ASHA areas were recruited for the second round, conducted between August and October 2016. Of the eligible respondents, 1770 (80%) were available and completed surveys. Comparisons of participants in the original and follow-up surveys revealed no demographic differences (i.e., attrition-related biases). Data for current analyses are taken from this second survey, as assessments of RC were not included in the first wave. Data were weighted based on the sampling design to produce estimates representative of the selected blocks within the 49 included districts of Uttar Pradesh.

Survey interviews that focused on health facility usage, family planning, and reproductive health outcomes were conducted by female research staff in a private area of the participants’ choosing in the immediate vicinity of their home. Consent material and all survey items were read aloud to participating women in Hindi. Study protocols were reviewed and approved by the National Rural Health Mission of Uttar Pradesh, Public Health Service—Ethical Review Board (PHS-ERB) - an independent ethical review board, and the Health Ministry Screening Committee of the Indian Council for Medical Research.

### Measures

The dependent variables in this analysis relate to use of modern contraceptives, unintended pregnancy, and pregnancy termination. Contraceptive use in the past year was defined as use of any modern contraceptive method in the past 12 months (modern methods include oral contraceptive pills, intrauterine devices, injectable contraception, male condoms, subdermal implants, diaphragms, lactational amenorrhea, and emergency contraceptive pills). Analyses involving this contraceptive use outcome included only those women who were not sterilized, whose husbands were not sterilized, and who were not currently pregnant at the time of the survey (n = 1424). Unintended pregnancy was assessed based on reported pregnancy intentions at the time that they most recently became pregnant: women were asked whether they wanted to become pregnant at that time, wanted to become pregnant but at a later date, or did not want to have any or any more children at that time. Those that indicated wanting to either become pregnant later or not at all were considered to have experienced an unintended pregnancy. Abortion in the past two years was assessed based on women's responses to the question “In the past two years, have you ever voluntarily terminated or aborted a pregnancy?”

Independent variables included ever experiencing RC by a current husband or in-laws, lifetime experience of physical IPV by a current husband, and lifetime experience of sexual IPV by a current husband. Lifetime RC was measured via eight questions related to women's experiences of coercion or force from husbands or in-laws regarding reproductive choices, including use of family planning methods. These items were drawn from an RC scale validated among US women (McCauley et al., 2017; Miller et al., 2014) and adapted for the Indian context. The RC scale was adapted based on formative research in the forms of interviews with women and health care providers in Maharashtra, India (Ghule et al., 2015), and group discussions with women's self-help groups across four communities in Uttar Pradesh (unpublished data). Adaptation followed a process recommended by Jose, Bhan, and Raj (2017). The equally weighted eight items refer to whether a woman's husband or in-laws had ever stopped her from going or refused to give permission for her to go to a clinic or community health event to get family planning; destroyed, hidden, or taken a family planning method (such as pills) away from her; told her that they would abandon her if she tried to prevent or delay getting pregnant; told her that she would be beaten if she tried to prevent or delay getting pregnant; told her that it was against their religion or culture to use family planning; told her that women who use family planning do this so that they can have sex with other men; told her that she could not use family planning because she did not have any or enough sons; or not permitted her to use contraceptives. Lifetime RC was indicated by a “yes” response to any of these items. This assessment of RC was found to be internally reliable among the current sample (Cronbach's alpha = 0.73). For women who reported one or more forms of RC, the perpetrator of these acts was assessed via a single item (Who did or said this?), with response choices of “husband,” “in-laws,” or “both husband and in-laws.”

Physical IPV was indicated by a positive response to whether the participant's current husband had ever slapped her, twisted her arm or pulled her hair; pushed her, shook her, or threw something at her; kicked her, dragged her, or beat her up; punched her with his fist or with something that could hurt her; or threatened or attacked her with a knife, gun, or any other weapon. Similarly, sexual IPV was indicated by a positive response to ever having experienced sexual aggression from her current husband: he physically forced her to have sexual intercourse with him when she did not want to; he physically forced her to perform other sexual acts she did not want to; he used threats or other actions to make her perform sexual acts when she did not want to; she had sexual intercourse when she did not want to because she was afraid of what her husband might do if she refused; he forced her to do something sexual that she found degrading or humiliating. Both physical and sexual IPV assessments were drawn from the WHO Multi-country Study on Women's Health and Domestic Violence (2005).

Sociodemographic measures included caste, religion, household wealth, literacy of woman, husband's education, age of woman, age of woman at first marriage, and birth parity. To capture both caste and religion-based marginalization, households were sorted into three caste/religion categories: Scheduled Caste/Scheduled Tribe (SC/ST; lowest castes), Muslim, and neither SC/ST nor Muslim. The Standard of Living Index (SLI) was used as a proxy indicator for characterizing household wealth (IIPS, 1999); the SLI methodology is used for this purpose in the Demographic and Health Surveys across multiple national contexts, including India (IIPS, 1999). SLI scores were sorted into quartiles based on scores of 0–25, 26 to 50, 51 to 75, and 76 to 100 (range 0–100) to create scores of 1 through 4 for level of household wealth. Regarding literacy, a woman was considered literate if she reported being able to both read and write in at least one language. Husband education was dichotomized based on reports of whether he had completed primary education in school (i.e., completed school through fifth grade).

### Analysis

Chi-square tests were used to evaluate the associations of demographics with key predictor variables (lifetime reproductive coercion [both overall and perpetrator-specific], lifetime physical IPV from a current husband, and lifetime sexual IPV from a current husband) and outcomes (modern contraceptive use in the past year, unintended pregnancy for most recent pregnancy, and abortion in the past two years). Chi-square tests were also performed to assess associations between these predictors and outcomes. Logistic regression models adjusted for demographics further described the relationships (adjusted odds ratio [aOR] and 95% confidence interval [CI]) between predictors and outcomes. Multivariate logistic regression models that included physical IPV, sexual IPV, and RC (one model inclusive of overall RC and one inclusive of perpetrator-specific RC) were developed to evaluate the independent contributions of each form of abuse to the reproductive health outcomes. Models were adjusted a priori for caste, religion, wealth index, literacy of woman, husband's education, age of woman, age of woman at first marriage, and birth parity based on their known associations with outcomes. Covariates were tested for multicollinearity. As described earlier, analyses inclusive of use of modern contraceptives in the past year excluded women who were sterilized, whose husbands were sterilized, or who were currently pregnant. Sample weights calculated based on the multistage sampling design were utilized in all analyses. Data were analyzed using STATA 12.0 software (StataCorp, USA).

## Results

Approximately 1 in 7 women (14%; 95% CI, 10.7–17.5) reported that their most recent pregnancy was unintended, and 1% reported having an abortion in the past 2 years (95% CI, 0.8–2.2) (Table 1). Among all women not currently pregnant (n = 1424), approximately 1 in 4 reported use of modern contraceptives (23%; 95% CI, 19.9–25.9) during the past 12 months. Women married prior to age 18 years were more likely than those who married at older ages to report that their most recent pregnancy was unintended, and literate women were more likely than illiterate women to report using modern contraception in the past year and less likely to report their last pregnancy as unintended. Unsurprisingly, women with higher numbers of children were more likely than those with fewer children to report their most recent pregnancy as unintended (all *p* values < 0.05).

**Table 1:** Frequencies of sample demographics by outcomes of interest.

	Total		FP Use - past 12 months			Unintended pregnancy - most recent pregnancy			Abortion - past 2 years		
					p-value			p-value			p-value
	Unwtd. N	%	Unwtd. N	%		Unwtd. N	%		Unwtd. N	%	
		(95% CI)		(95% CI)			(95% CI)			(95% CI)	
Total	1770		362	23%		277	14%		33	1%	
				(19.9–25.9)			(10.7–17.5)			(.8–2.2)	
Background characteristics											
Age											
15-19	4	0.5%	0	0.00%	0.68	1	32%	0.02	0	0%	0.88
		(0.2–1.4)					(3.9–83.9)				
20-24	193	9%	47	22%		19	6%		5	2%	
		(6.9–11.3)		(14.2–31.9)			(3.2–11.1)			(.7–5.7)	
25-29	345	18%	84	21%		52	9%		8	2%	
		(14.6–20.9)		(15.9–28.2)			(5.8–14.9)			(.6–4.5)	
30+	1228	73%	231	24%		205	16%		20	1%	
		(69.5–76.3)		(19.8–27.7)			(12.0–20.0)			(.7–2.3)	
Age at marriage											
<18	1609	90%	321	23%	0.91	261	15%	0.01	27	1%	0.09
		(87.5–92.4)		(19.4–26.3)			(11.4–18.6)			(.7–2.1)	
18+	161	10%	41	23%		16	6%		6	3%	
		(7.5–12.4)		(15.1–34.2)			(2.6–12.1)			(1.2–6.8)	
Wealth quartile											
1 (poorest)	374	19%	66	21%	0.05	55	15%	0.50	3	1%	0.11
		(15.6–22.7)		(15.4–27.7)			(9.6–21.4)			(.3–5.4)	
2	695	36%	128	20%		111	16%		15	2%	
		(31.8–40.8)		(15.9–23.9)			(10.9–21.8)			(1.3–4.2)	
3	589	37%	135	24%		96	13%		13	1%	
		(32.6–41.7)		(18.3–31.1)			(8.5–18.6)			(0.3–1.5)	
4 (wealthiest)	112	8%	33	35%		15	8%		2	0%	
		(5.7–10.5)		(25.2–46.5)			(3.5–18.6)			(0.1–1.7)	
Literacy											
Illiterate	1223	69%	218	20%	0.03	192	14%	0.42	24	2%	0.29
		(66.2–72.4)		(16.9–23.9)			(10.8–18.7)			(0.9–2.7)	
Literate	547	31%	144	28%		85	12%		9	1%	
		(27.6–33.8)		(22.4–33.9)			(8.9–17.2)			(0.4–2.1)	
Spouse literacy	(Proxy for literacy:attended school till 5th/completed primary school)										
Illiterate	574	32%	99	20%	0.19	99	19%	0.02	12	2%	0.16
		(28.7–36.4)		(15.9–24.9)			(13.0–25.8)			(0.9–4.4)	
Literate	1196	68%	263	24%		178	11%		21	1%	
		(63.6–71.3)		(20.3–28.4)			(8.4–15.4)			(0.6–1.8)	
Caste/religion											
Neither SC/ST nor Muslim	1103	62%	233	25%	0.19	167	13%	0.57	21	1%	0.91
		(57.4–66.3)		(20.5–29.3)			(9.5–16.9)			(0.7–2.6)	
SC/ST	400	22%	79	23%		59	15%		8	1%	
		(18.6–26.1)		(17.2–29.0)			(8.9–22.8)			(0.6–3.2)	
Muslim	267	16%	50	17%		51	17%		4	1%	
		(12.0–20.7)		(12.3–24.3)			(10.3–25.7)			(0.3–3.3)	
Parity											
0	212	12%	53	23%	0.54	19	5%	0.00	6	2%	0.25
		(9.2–14.3)		(15.4–32.5)			(2.7–10.6)			(0.9–5.7)	
1	152	7%	43	28%		13	8%		5	3%	
		(5.6–9.5)		(19.6–38.9)			(3.6–17.8)			(0.9–7.4)	
2	275	14%	63	25%		36	11%		7	1%	
		(12.4–16.8)		(17.8–34.7)			(6.4–17.3)			(0.5–3.2)	
3+	1131	67%	203	21%		209	16%		15	1%	
		(63.4–69.8)		(17.8–25.2)			(12.6–21.2)			(0.5–2.2)	

More than 1 in 3 women reported ever experiencing physical IPV (36%), and 8% reported ever experiencing sexual IPV from their current husband (Table 2). Approximately 1 in 8 (12%) women reported ever experiencing RC from their current husbands or in-laws; 43% of these women reported RC by husbands only, 49% reported RC by in-laws only, and 10% reported RC by both husbands and in-laws. Of those women reporting RC, 54% also reported physical IPV and 8% reported sexual IPV.

**Table 2:** Numbers and percentages of reproductive health outcomes among women in Uttar Pradesh, India based on expereicnes of reproductive coercion, reproductive coercion by perpetrator, physical IPV and sexual IPV.

	Total		Recent FP Use (n = 1424)			Unintended Pregnancy (n = 1770)			Abortion in past two years (n = 1770)		
	Unwtd. N	%	Unwtd. N	%	p-value	Unwtd. N	%	p-value	Unwtd. N	%	p-value
		(95% CI)	362	(95% CI)		277	(95% CI)		33	(95% CI)	
*Predictors*											
Reproductive Coercion											
No	1556	87.7%	333	25.7%	0.00	202	10.6%	0.00	28	1.4%	0.99
		(84.5–90.3)		(22.3–29.3)			(8.2–13.7)			(0.8–2.2)	
Yes	214	12.3%	29	6.4%		75	36.1%		5	1.4%	
		(9.6–16)		(3.5–11.6)			(23.6–47.9)			(0.4–4.0)	
Reproductive Coercion Perpetrator											
Husband Only	91	4.8%	7	5.6%	0.00	38	38.1%	0.00	2	1.3%	0.57
		(3.5–6.5)		(2.2–13.3)			(25.1–53.0)			(0.8–2.2)	
In-laws Only	104	5.9%	22	8.8%		34	38.9%		3	2.40%	
		(4.1–8.3)		(3.7–19.6)			(21.8–59.4)			(0.6–8.4)	
Both	21	1.6%	0	0.0%		3	17.3%		0	0	
		(0.9–2.9)					(4.2–49.9)				
Husband Physical IPV											
No	1114	63.6%	239	24.9%	0.03	113	7.4%	0.00	19	0.6%	0.00
		(58.1–68.7)		(21.2–29.1)			(5.5–9.8)			(0.3–1.2)	
Yes	656	36.4%	123	19.1%		164	24.9%		14	2.5%	
		(31.2–41.8)		(15.6–23.3)			(18.5–32.6)			(1.4–4.6)	
Husband Sexual IPV											
No	1621	91.5%	332	23.4%	0.09	246	13.7%	0.80	29	1.3%	0.48
		(88.7–93.7)		(20.4–26.7)			(10.5–17.6)			(0.8–2.1)	
Yes	149	8.4%	30	15.7%		31	14.6%		4	2.3%	
		(6.3–11.2)		(9.7–24.6)			(8.6–23.6)			(0.5–11.2)	

In unadjusted logistic regressions, women who had experienced physical IPV were significantly more likely to report their most recent pregnancy as unintended (OR, 4.3; 95% CI, 2.8–6.5) and to have had an abortion in the past 2 years (OR, 3.4; 95% CI, 1.4–8.5) than women who had not experienced physical IPV (Table 3). Women who had experienced RC had significantly lower odds of having used modern contraception in the past year (OR, 0.2; 95% CI, 0.1–0.4) and higher odds of their most recent pregnancy being unintended (OR, 4.7; 95% CI, 2.7–8.3) than their peers who had not been exposed to RC. When broken down by perpetrator type for RC, experiencing RC perpetrated by husbands only or by in-laws only conferred similar reduced odds of recent contraceptive use (husband only: OR 0.2, 95% CI 0.1–0.5; in-laws only: OR 0.3, 95% CI 0.1–0.7) and increased odds of unintended pregnancy (husband only: OR 5.2, 95% CI 2.7–10.2; in-laws only: OR 5.4, 95% CI 2.4–12.3). Sexual IPV was not associated with any of the three reproductive outcomes assessed.

**Table 3:** Unadjusted logistic regression models of reproductive coercion, reproductive coercion by perpetrator, physical IPV, and sexual IPV as predictors of reproductive health outcomes.

		Recent FP Use	Unintended Pregnancy	Abortion in last two years
		(n = 362)	(n = 277)	(n = 33)
		OR (95% CI)	OR (95% CI)	OR (95% CI)
Lifetime Reproductive Coercion	No	REF	REF	REF
	Yes	0.18 (0.09–0.36)***	4.70 (2.65–8.31)***	0.87 (0.23–3.34)
Reproductive Coercion Perpetrator	No RC	REF	REF	REF
	Husband Only	0.16 (0.06–0.45)**	5.22 (2.67–10.18)***	0.31 (0.06–1.56)
	In-laws Only	0.26 (0.10–0.68)**	5.43 (2.39–12.29)***	1.45 (0.31–6.77)
Lifetime Husband Physical IPV	No	REF	REF	REF
	Yes	0.72 (0.51–1.01)	4.29 (2.82–6.53)***	3.44 (1.40–8.46)**
Lifetime Husband Sexual IPV	No	REF	REF	REF
	Yes	0.62 (0.33–1.15)	1.03 (0.55–1.91)	2.15 (0.32–14.25)

**Statistically significant at p < 0.05.

***Statistically significant at p < 0.001.

Multivariate logistic regression models adjusted for covariates and inclusive of all three forms of violence yielded findings consistent with unadjusted analyses, supporting the premise that RC and IPV not only relate to reproductive outcomes differently but also independently of each other (Table 4). Women who experienced physical IPV had 4.3 (95% CI, 2.7–6.8) times greater odds of having their most recent pregnancy be unintended and 3.4 (aOR; 95% CI, 1.4–8.7) times greater odds of having had an abortion in the past two years than women who did not experience physical IPV, independent of the effects of experiencing RC and sexual IPV. Women who experienced RC were 80% less likely (aOR, 0.2; 95% CI, 0.1–0.4) to have recently used modern contraception and had approximately 4 times greater odds (aOR, 3.9; 95% CI, 2.3–6.7) of reporting their most recent pregnancy as unintended than women who reported no RC experiences; neither physical nor sexual IPV were associated with recent family planning in the presence of RC. As in unadjusted analyses, RC perpetrated by husbands and in-laws predicted these same outcomes at similar magnitudes.

**Table 4:** Adjusted multivariate logistic regression models of reproductive coercion, physical IPV and sexual IPV as predictors of reproductive health outcomes.

		Recent FP Use^a^	Unintended Pregnancy^b^	Abortion in last two years^c^
		(n = 362)	(n = 277)	(n = 33)
		AOR (95% CI)	AOR (95% CI)	AOR (95% CI)
Lifetime Reproductive Coercion	No	REF	REF	REF
	Yes	0.19 (0.09–0.37)***	3.91 (2.27–6.74)***	0.70 (0.2–2.7)
Lifetime Husband Physical IPV	No	REF	REF	REF
	Yes	0.86 (.60–1.22)	4.32 (2.74–6.80)***	3.42 (1.35–8.66)**
Lifetime Husband Sexual IPV	No	REF	REF	REF
	Yes	0.63 (0.32–1.23)	0.53 (0.27–1.03)	1.24 (0.19–8.11)

**Statistically significant at p < 0.05.

***Statistically significant at *p* < 0.001.

a No other variables retained statistical significance in the final model.

b Literacy and parity remained associated with recent unintended pregnancy in the final model.

c Age at marriage remained associated with recent abortion in the final model.

## Discussion

In this first population-based study of RC (i.e., behaviors that directly interfere with a woman's wishes and attempts to prevent becoming pregnant) from male partners and in-laws in an LMIC setting, approximately 1 in 8 women in Uttar Pradesh reported experiencing this form of gender-based violence. RC was reported to be perpetrated by both husbands and in-laws in this context, at similar frequencies. Thus, RC appears to be a common experience among this high-need population and should be conceptualized as a form of family violence not restricted to husbands. Indeed, almost half of all RC cases identified would have remained undetected if our assessments were limited to husband perpetration. These findings of both husband and in-law RC perpetration echo those found in recent studies of other forms of gender-based family abuse and coercion (e.g., those examining gender-based household maltreatment) (Silverman et al., 2016a, 2016b).

Among women experiencing RC, more than one-third (36%) reported that their most recent pregnancy was unintended; these women had 4 to 5 times greater odds of unintended pregnancy than women not experiencing RC, after accounting for effects of demographics and physical and sexual IPV. Similar to findings of previous studies of IPV in India, physical IPV also greatly increased the risk of unintended pregnancy (Stephenson et al., 2008; Begum, Dwivedi, Pandey, & Mittal, 2010; Yoshikawa, Agrawal, Poudel, & Jimba, 2012; Raj & McDougal, 2015). The independent nature of these effects indicates that both RC and IPV relate to reduced reproductive control and that these risks are distinct from one another. This has critical implications for both community and clinic-based interventions seeking to reduce women's risk of unintended pregnancy, in that effective programs may require directly addressing both RC and IPV.

Experiences of reproductive coercion were also associated with a more than 5 times decreased likelihood of women's recent use of modern contraceptives, with only 6% of women reporting RC using such family planning in the past year; IPV was not a significant predictor of contraceptive use after accounting for the effects of RC. Similar to previous studies of RC, such coercion (whether by husbands or in-laws in this context, as no differences in effects were found based on relationship) appears to make it more difficult for married women in Uttar Pradesh to access or use effective forms of contraceptives, with subsequent increased risk for unintended pregnancy (Gupta, Falb, Kpebo, & Annan, 2012; Miller et al., 2010; Miller et al., 2014; Silverman et al., 2007). This result suggests that community-based interventions and health workers may need to recognize and address RC as a barrier to access and that health systems and clinic-based providers offering family planning must make similar efforts to increase use of effective contraceptives among the many women experiencing these forms of coercion. Specifically, efforts to assist women experiencing RC should educate and provide clear counselling to such women regarding identifying contraceptive methods that may be used without partner or in-law knowledge (e.g., intrauterine devices). Current guidelines to address IPV in healthcare settings, including those involving provision of contraceptives, do not currently include assessment or addressing of RC (Bhawan, 2013a, Bhawan, 2013b; WHO 2014).

Experiences of IPV, and not RC, were associated with women's reports of abortion in the past two years. This may suggest that, although both RC and IPV contribute to unintended pregnancy, physical violence from husbands may drive decisions (coerced or otherwise) to terminate such pregnancies. The finding of a positive association between IPV and abortion is consistent with previous research (Pallitto et al., 2013). Reasons for this may include women's fear of their ability to cope with or take care of another child in the context of ongoing physical abuse (Chibber et al., 2014). As IPV has been associated with son preference in the Indian context, this increased prevalence of abortions may also be prompted by sex determination indicating that the fetus is female (Jha et al., 2006) and the consequent aversion to having a (or another) girl child in households that consider women and girls to be of lower worth (Puri, Adams, Ivey, & Nachtigall, 2011; Sabarwal, McCormick, Subramanian, & Silverman, 2012; Silverman et al., 2011). The absence of an association between sexual IPV and unintended pregnancy is consistent with recent analyses of national data from India (Raj & McDougal, 2015). Regarding the lack of association between sexual IPV and contraceptive use, recent multinational analyses of data from across South Asia indicate that associations between contraceptive use and sexual IPV are method specific, with sexual IPV positively associated with use of some methods of contraception but negatively associated with others (Raj, McDougal, Reed, & Silverman, 2015). Current analyses were not method specific, and thus may have masked such effects.

### Limitations

Major limitations of the design of the current study include the use of cross-sectional data, constraining any inferences regarding causality or ordering of events. Related to this same concern, the current analyses employed data on lifetime IPV and RC victimization to model outcomes of contraceptive use in the past 12 months, recent unintended pregnancy, and pregnancy termination in the past 24 months. The lack of congruency in these retrospective timeframes further limits our ability to make conclusions regarding the nature of observed associations.

## Conclusion

In sum, RC is a prevalent and critical barrier to women's reproductive autonomy in the populous and high-need state of Uttar Pradesh in India. The current study indicates that the 1 in 8 women who experience RC are at significantly greater risk of poor reproductive health outcomes, including unintended pregnancy and contraceptive nonuse, and that these effects are independent of and beyond those that may be attributed to IPV. Scalable and sustainable interventions to reduce RC in clinical and community settings are likely key to improving women's reproductive autonomy and health in India and globally.

## Funding disclosure

This study was funded by the Bill and Melinda Gates Foundation Grant No. OPP1083531.

I wish to confirm that there are no known conflicts of interest associated with this publication and there has been no significant financial support for this work that could have influenced its outcome.

## Declaration of competing interest

None.

## References

[bib1] Ahmed S., Creanga A.A., Gillespie D.G., Tsui A.O. (2010). Economic status, education and empowerment: Implications for maternal health Service utilization in developing countries. PLoS One.

[bib2] Ahmed S., Li Q., Liu L., Tsui A.O. (2012). Maternal deaths averted by contraceptive use: An analysis of 172 countries. Lancet.

[bib3] Barua A., Kurz K. (2001). Reproductive health-seeking by married adolescent girls in Maharashtra, India. Reprod. Health Matters.

[bib4] Begum S., Dwivedi S.N., Pandey A., Mittal S. (2010). Association between domestic violence and unintended pregnancies in India: Findings from the national family health survey-2 data. The National Medical Journal of India.

[bib5] Bhawan N. (2013). Guidance note for implementation of RMNCH+A interventions in high priority districts. National rural health mission.

[bib6] Bhawan N. (2013). Guidelines and protocols medico-legal care for survivors/victims of sexual violence.

[bib7] Chibber K.S., Biggs M.A., Roberts S.C., Foster D.G. (2014 Jan 1). The role of intimate partners in women's reasons for seeking abortion. Women's Health Issues.

[bib8] Cripe S.M., Sanchez S.E., Perales M.T., Lam N., Garcia P., Williams M.A. (2008). Association of intimate partner physical and sexual violence with unintended pregnancy among pregnant women in Peru. International Journal of Gynecology & Obstetrics.

[bib9] Fernandez M. (1997). Domestic violence by extended family members in India. Journal of Interpersonal Violence.

[bib10] Filippi V., Ronsmans C., Campbell O.M.R., Graham W.J., Mills A., Borghi J. (2006). Maternal health in poor countries: The broader context and a call for action. Ser. Matern. Surviv.

[bib11] Gao W., Paterson J., Carter S., Iusitini L. (2008). Intimate partner violence and unplanned pregnancy in the pacific islands families study. International Journal of Gynecology & Obstetrics.

[bib12] Ghule M., Raj A., Palaye P., Dasgupta A., Nair S., Saggurti N. (2015). Barriers to use contraceptive methods among rural young married couples in Maharashtra, India: Qualitative findings. Asian Journal of Research in Social Sciences and Humanities.

[bib13] Government of India, National Heath Mission (2017). District guidelines, reducing maternal mortality. https://darpg.gov.in/sites/default/files/National%20Health%20Mission.pdf.

[bib14] Government of India, National Heath Mission (2018). Family planning schemes, guidelines and important government orders. http://www.nhm.gov.in/nrhm-components/rmnch-a/family-planning/schemes,-guidelines-important-government-orders.html.

[bib15] Gupta J., Falb K., Kpebo D., Annan J. (2012). Abuse from in-laws and associations with attempts to control reproductive decisions among rural women in côte d'Ivoire: A cross-sectional study. BJOG: An International Journal of Obstetrics and Gynaecology.

[bib16] Indicators from Annual Health Survey (2012-2013).

[bib17] International Institute for Population Sciences (IIPS) (1999).

[bib18] International Institute for Population Sciences (IIPS) and ICF (2017). National family health survey (NFHS-4).

[bib19] International Institute for Population Sciences (IIPS) and ICF (2017). I.

[bib20] Jha P., Kumar R., Vasa P., Dhingra N., Thiruchelvam D., Moineddin R. (2006). Low male-to-female sex ratio of children born in India: National survey of 1· 1 million households. The Lancet.

[bib21] Jose R., Bhan N., Raj A. (2017). EMERGE measurement guidelines report 2: How to create scientifically valid social and behavioral measures on gender equality and empowerment. Center on gender equity and health (GEH), university of California, san diego school of medicine. San diego, CA. November.

[bib22] Kazmerski T., McCauley H.L., Jones K., Borrero S., Silverman J.G., Decker M.R. (2015). Use of reproductive and sexual health services among female family planning clinic clients exposed to partner violence and reproductive coercion. Maternal and Child Health Journal.

[bib23] McCauley H.L., Silverman J.G., Jones K.A., Tancredi D.J., Decker M.R., McCormick M.C. (2017). Psychometric properties and refinement of the reproductive coercion scale. Contraception.

[bib24] Miller E., Decker M.R., McCauley H.L., Tancredi D.J., Levenson R.R., Waldman J. (2010). Pregnancy coercion, intimate partner violence and unintended pregnancy. Contraception.

[bib25] Miller E., McCauley H.L., Tancredi D.J., Decker M.R., Anderson H., Silverman J.G. (2014). Recent reproductive coercion and unintended pregnancy among female family planning clients. Contraception.

[bib26] Miller E., Tancredi D.J., Decker M.R., McCauley H.L., Jones K.A., Anderson H. (2016). A family planning clinic-based intervention to address reproductive coercion: A cluster randomized controlled trial. Contraception.

[bib27] Mishra A., Nanda P., Speizer I.S., Calhoun L.M., Zimmerman A., Bhardwaj R. (2014). Men's attitudes on gender equality and their contraceptive use in Uttar Pradesh India. Reproductive Health.

[bib28] Moore A.M., Frohwirth L., Miller E. (2010). Male reproductive control of women who have experienced intimate partner violence in the United States. Social Science & Medicine.

[bib29] Pallitto C.C., García-Moreno C., Jansen H.A.F.M., Heise L., Ellsberg M., Watts C. (2013). WHO multi-country study on women's health and domestic violence.

[bib30] Puri S., Adams V., Ivey S., Nachtigall R.D. (2011). “There is such a thing as too many daughters, but not too many sons”: A qualitative study of son preference and fetal sex selection among Indian immigrants in the United States. Social Science & Medicine.

[bib31] Raj A., McDougal L. (2015). Associations of intimate partner violence with unintended pregnancy and pre-pregnancy contraceptive use in South Asia. Contraception.

[bib32] Raj A., McDougal L., Reed E., Silverman J.G. (2015 Aug 1). Associations of marital violence with different forms of contraception: Cross-sectional findings from South Asia. International Journal of Gynecology & Obstetrics.

[bib33] Raj A., Sabarwal S., Decker M.R., Nair S., Jethva M., Krishnan S. (2011). Abuse from in-laws during pregnancy and post-partum: Qualitative and quantitative findings from low-income mothers of infants in Mumbai, India. Maternal and Child Health Journal.

[bib34] Sabarwal S., McCormick M.C., Subramanian S.V., Silverman J.G. (2012). Son preference and intimate partner violence victimization in inida: Examining the role of actual and desired family composition. Journal of Biosocial Science.

[bib35] Silverman J.G., Balaiah D., Decker M.R., Boyce S.C., Ritter J., Naik D.D. (2016). Family violence and maltreatment of women during the perinatal period: Associations with infant morbidity in Indian slum communities. Maternal and Child Health Journal.

[bib36] Silverman J.G., Balaiah D., Ritter J., Dasgupta A., Boyce S.C., Decker M.R. (2016). Maternal morbidity associated with violence and maltreatment from husbands and in-laws: Findings from Indian slum communities. Reproductive Health.

[bib37] Silverman J.G., Decker M.R., Cheng D.M., Wirth K., Saggurti N., McCauley H.L. (2011). Gender-based disparities in infant and child mortality based on maternal exposure to spousal violence. Archives of Pediatrics and Adolescent Medicine.

[bib38] Silverman J., Gupta J., Decker M., Kapur N., Raj A. (2007). Intimate partner violence and unwanted pregnancy, miscarriage, induced abortion, and stillbirth among a national sample of Bangladeshi women. BJOG: An International Journal of Obstetrics and Gynaecology.

[bib39] Stephenson R., Koenig M.A., Acharya R., Roy T.K. (2008). Domestic violence, contraceptive use, and unwanted pregnancy in rural India. Studies in Family Planning.

[bib40] Tancredi D.J., Silverman J.G., Decker M.R., McCauley H.L., Anderson H.A., Jones K.A. (2015). Cluster randomized controlled trial protocol: Addressing reproductive coercion in health settings (ARCHES). BMC Women's Health.

[bib41] WHO Health care for women subjected to intimate partner violence or sexual violence (2014). A clinical handbook.

[bib42] WHO Multi-country Study on Women’s Health and Domestic Violence against Women (2005). Summary report.

[bib43] WHO Strengthening health systems to respond to women subjected to intimate partner violence or sexual violence (2017). A manual for health managers.

[bib44] WHO UN Sustainable Development Summit (2015).

[bib45] Yoshikawa K., Agrawal N.R., Poudel K.C., Jimba M. (2012). A lifetime experience of violence and adverse reproductive outcomes: Findings from population surveys in India. Biosci. Trends.

